# Fatty acid composition in serum lipids and the renal function of type 2 diabetes mellitus patients

**DOI:** 10.1186/1758-5996-7-S1-A35

**Published:** 2015-11-11

**Authors:** Ana Luiza Teixeira dos Santos, Cristina Pavinatto, Magda Perassolo, Camila Kümmel Duarte, Maira Zoldan, Lorenzo Catucci Boza, Mirela Jobim de Azevedo, Jorge Luiz Gross, Themis Zelmanovitz

**Affiliations:** 1 HCPA, Porto Alegre, Brazil

## Background

Dietary and serum fatty acids composition have been related to Diabetic Kidney Disease. Not only this subject is still controversial, but also most of the studies focus on the association with albuminuria. Regarding the relation of serum fatty acids with glomerular filtration rate (GFR), the data are scarce.

## Objective

To evaluate the possible association of albuminuria and glomerular filtration rate with serum fatty acids composition of Type 2 diabetes patients, with and without Diabetic Kidney Disease.

## Materials and methods

In this cross-sectional study, the patients were submitted to nutritional, clinical and laboratory evaluation, emphasizing the diabetic chronic complications. Serum fatty acids composition in total lipids was measured by gas chromatography. Albuminuria (24-hour urinary albumin excretion [UAE]) was measured twice and glomerular filtration rate (GFR) was estimated by using the CKD-EPI equation.

## Results

A total of 128 patients were evaluated (66 [51.6%] male, mean age 60±10 yrs., duration of diabetes 10±7 yrs., body mass index 28.5±4.3 kg/m^2^, median UAE 11 (3-843) mg/24-h and mean eGFR 95±18 ml/min/m2). In multiple linear regression models, adjusting for using hypolipidemic agents and ACE inhibitors and/or angiotensin receptor blockers, 24-hour UAE was inversely associated with serum levels of total polyunsaturated fatty acids (R2=0.067, β -Standardized Coefficients=- 0.196; P=0.030). On the other hand, in another model adjusting for age and using hypolipidemic agents, eGFR was positively associated with serum levels of saturated fatty acids (R2=0.250, β -Standardized Coefficients=0.238; P=0.004) and negatively associated with serum polyunsaturated fatty acids (R2=0.234, β -Standardized Coefficients=- 0.199; P=0.014), especially linoleic acid (18: 2 n-6) (R2=0.250, β -Standardized Coefficients=- 0.237; P=0.003).

## Conclusion

In type 2 diabetes patients, serum polyunsaturated fatty acids were inversely associated with albuminuria. In contrast, these fatty acids, especially linoleic acid might be deleterious to eGFR. Surprisingly, serum saturated fatty acids was positively associated to eGFR in these patients. More studies are needed to clarify these findings.

